# Bridging neural circuits and clinical outcomes: a novel framework for perioperative disturbances of consciousness and cognition

**DOI:** 10.3389/fneur.2026.1734294

**Published:** 2026-03-24

**Authors:** Xiangzhen Wang, Xiaojia Ma, Ziying Shen, Yuanfang Jia, Yanting Wang, Nannan Zhang

**Affiliations:** 1 Department of Anaesthesiology, Affiliated Hospital of Qingdao University Medical College, Qingdao, Shandong, China; 2 Department of Social Prevention and Control, Qingdao Mental Health Center, Qingdao, Shandong, China

**Keywords:** anesthesia, anesthetic depth monitoring, multimodal monitoring, neural circuits, perioperative disturbances of consciousness and cognition

## Abstract

Disturbances of consciousness and cognition that happen during the perioperative period remain a critical challenge in anesthesiology, manifested as intraoperative awareness, delayed emergence, and postoperative delirium. These disorders arise from complex interactions among neural connectivity, neurotransmitter dynamics, and the pharmacological modulation of cortical and subcortical circuits. The thalamocortical network, brainstem arousal centers, and frontoparietal integration systems are crucial in facilitating transitions between drug-induced unconsciousness and recovery; however, their functional disruptions are only partially comprehended. This paper synthesizes recent research that connecting consciousness neurobiology with anesthetic mechanisms, examining how dysregulated GABAergic, NMDA, and adrenergic signaling underlies perioperative neural states. It examines the dual spectrum of intraoperative awareness and postoperative cognitive disturbances through a comprehensive neurophysiological framework. Furthermore, novel diagnostic technologies, including EEG-based depth indices, functional near-infrared spectroscopy, and AI-assisted models, are analyzed for their potential in the real-time detection and prediction of states of consciousness. This study seeks to establish a comprehensive framework for understanding perioperative disturbances of consciousness and cognition by integrating mechanistic, clinical, and technological perspectives. It stresses the importance of personalized anesthetic approaches, multimodal monitoring, and neuroinformatics-informed interventions to avert cognitive sequelae and enhance neural recovery. This perspective positions anesthesiology at the intersection of neuroscience, data science, and consciousness research.

## 1 Introduction

Maintaining and regulating consciousness during anesthesia remains a major challenge in modern medicine. Anesthesiology has developed into a data-driven field that prioritizes accuracy, safety, and neuroprotection. However, the ongoing presence of perioperative disturbances of consciousness and cognition remains a significant challenge for both anesthesiologists and neuroscientists. The regulation of consciousness becomes particularly challenging when hemodynamics is unstable, as hemodynamic fluctuations can directly impact cerebral perfusion and alter anesthetic drug delivery to neural targets, compromising the delicate balance between adequate anesthesia and patient safety. These disturbances range from intraoperative awareness to postoperative delirium and cognitive dysfunction, reflecting interactions among neurobiology, pharmacology, and clinical monitoring. The capacity to reliably manipulate consciousness without residual disruption embodies the fundamental principles of anesthesia; however, it remains an incomplete science, in part due to the complex neurophysiological foundations of awareness and memory formation (1, 2).

Consciousness arises from distributed brain networks and can be reversibly modulated by anesthetic agents, yet the precise neurophysiological transitions remain incompletely understood (3). Although improved monitoring and anesthetic techniques have reduced the global incidence of intraoperative awareness with explicit recall, this complication still causes significant psychological effects, including post-traumatic stress disorder and enduring cognitive changes. These conditions not only extend hospital stays but also elevate morbidity, mortality, and long-term neurocognitive deterioration. The multifactorial characteristics of these disorders necessitate an integrative research paradigm that connects molecular neuroscience with real-time clinical observation (4, 5).

Functional magnetic resonance imaging (fMRI), electroencephalography (EEG), and functional near-infrared spectroscopy (fNIRS) have facilitated comprehensive mapping of brain connectivity across different anesthesia depths. These tools, along with machine learning algorithms, are now being used to figure out how deep an anesthetic is and to find early signs of waking up from an unconscious state. This is the first step toward precision-guided anesthetic management (6). In the field of pharmacology, the creation of anesthetic agents has moved from choosing them based on experience to designing them based on molecular information. Even though they work, the risk of perioperative consciousness disturbances is still affected by differences in metabolism, receptor sensitivity, and genetic predisposition between people. These variations highlight the necessity for personalized anesthesia, which involves customizing pharmacologic dosing and monitoring thresholds to specific neurophysiological responses, moving beyond the limitations of population-based dosing guidelines despite current intraoperative monitoring recommendations that primarily guide anesthetic depth rather than individual neurophysiological vulnerability (7, 8).

The moral consequences of disrupting and restoring consciousness are equally significant. These disturbances carry profound ethical and clinical implications, reinforcing the need for reliable prevention and recovery of consciousness. As a result, anesthesiology is no longer limited to inducing drug-induced unconsciousness; it has evolved to protect the integrity of cognitive recovery (9).

As the world’s population gets older and surgeries get more complicated, the number of people with perioperative disturbances of consciousness and cognition and how important they are to doctors is likely to go up. This makes it even more important for anesthesiologists to use a systems-based approach that combines neurobiology, artificial intelligence, and clinical analytics. The following chapters of this paper seek to dismantle these interconnected dimensions by examining the neurobiological foundations of consciousness, the pharmacodynamic principles that regulate anesthetic modulation, and the technological advancements that facilitate real-time evaluation of awareness. To address the multifactorial nature of perioperative disturbances of consciousness and cognition, we propose a triadic framework that integrates three interacting domains: (1) Neurobiological Substrate Layer: encompassing thalamocortical connectivity, neurotransmitter dynamics (GABAergic, NMDA, adrenergic), and receptor-level modifications that constitute the mechanistic core; (2) Clinical Phenotype Layer: comprising the observable manifestations of intraoperative awareness, delayed emergence, and postoperative delirium/cognitive dysfunction, each with distinct but overlapping risk trajectories; and (3) Technological Interface Layer: comprising EEG-derived indices, AI-assisted predictive models, and closed-loop delivery systems that translate neurobiological signals into real-time clinical decisions. This framework posits that consciousness disruption emerges from dynamic interactions across these layers, rather than from isolated pathophysiology, thereby guiding personalized anesthetic strategies that target specific neural vulnerabilities while monitoring cumulative cognitive risk. The subsequent sections will map empirical evidence onto this structure, culminating in a synthesis that operationalizes these principles for clinical implementation. Within this framework, neurobiological vulnerability is expressed as distinct clinical phenotypes and is imperfectly captured by technological surrogates, emphasizing that monitoring tools inform—but do not determine—clinical judgment.

## 2 Search methodology

**Figure 1:**
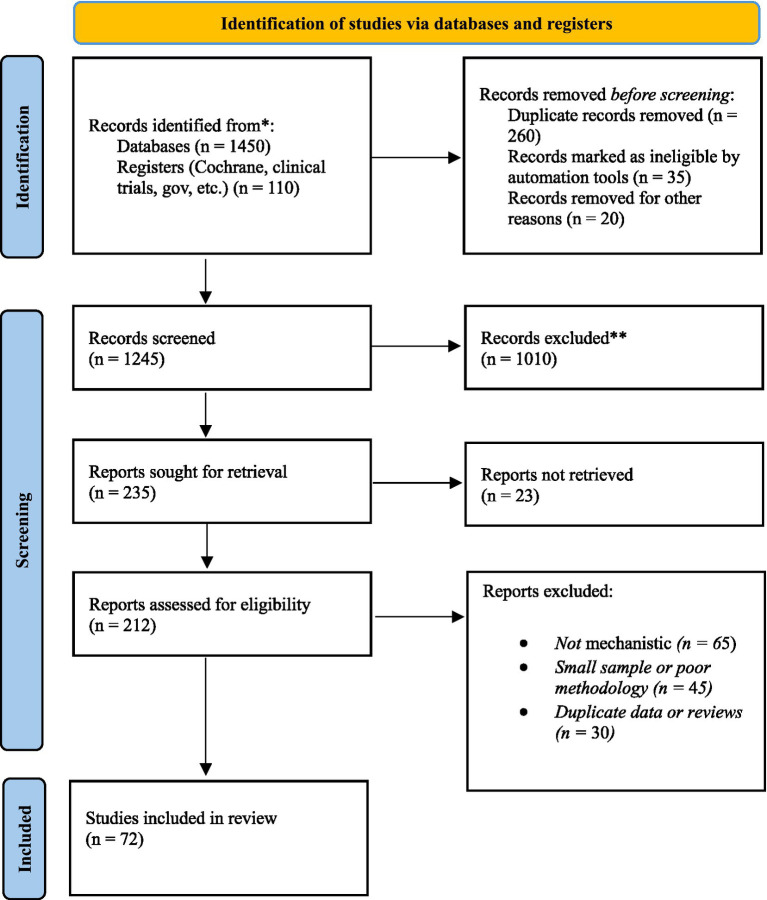
PRISMA flowchart.

The literature foundation for this study was established through a thorough and methodical search across various academic databases, including PubMed, Scopus, Web of Science, ScienceDirect, and SpringerLink, encompassing research published from January 2015 to September 2025. This time frame was carefully chosen to include the most recent and methodologically sound progress in anesthesiology, neuroscience, and cognitive research before, during, and after surgery. To make the search more precise and broad, a mix of controlled vocabulary and free-text keyword searches was used. To improve database retrieval, Boolean operators were used to search for terms like “perioperative consciousness disorders,” “intraoperative awareness,” “anesthetic depth monitoring,” “postoperative delirium,” “neurocognitive recovery,” “EEG under anesthesia,” “emergence phenomena,” and “anesthetic neurobiology.” Along with electronic searches, we also looked through reference lists of important reviews and meta-analyses by hand to find any publications that might have been missed by the algorithms. Only peer-reviewed studies that provided empirical, mechanistic, or clinical insights into consciousness modulation during anesthesia were included, with a preference for those demonstrating methodological rigor and reproducibility. To keep academic integrity and validity, we did not include non-scientific commentaries, isolated case reports, or materials that had not been peer-reviewed. We used Mendeley and Zotero software to organize and manage references. This made it possible to group them by theme into neurobiological, pharmacological, clinical, and technological domains. A total of 72 references were finalized for inclusion in this paper, each chosen for its scientific validity, relevance, and contribution to the comprehension of perioperative disturbances of consciousness and cognition phenomena. This structured methodological framework guarantees that the analysis in the following chapters is based on up-to-date, interdisciplinary, and evidence-based research, establishing a solid basis for higher-level interpretation and theoretical integration in anesthesiology (Figure 1).

## 3 Neurobiological mechanisms of consciousness

Consciousness is an emergent property of distributed neural networks, maintained by coordinated interactions across cortical and subcortical systems. General anesthesia disrupts this large-scale network coherence, producing a reversible state of unresponsiveness, although such disruption may be incomplete or unstable in certain perioperative conditions (1, 10, 11).

The thalamus is a central hub that controls the two-way flow of information between sensory cortices and higher-order association areas. While under general anesthesia, these circuits show less effective connectivity, which is shown by less thalamocortical synchrony and changes in gamma oscillations (12, 13). However, not all anesthetic agents impact these pathways consistently. Certain agents, including ketamine, elicit dissociative unconsciousness characterized by active sensory processing while perceptual integration disintegrates, underscoring the presence of various neurophysiological pathways to drug-induced unconsciousness.

**Figure 2:**
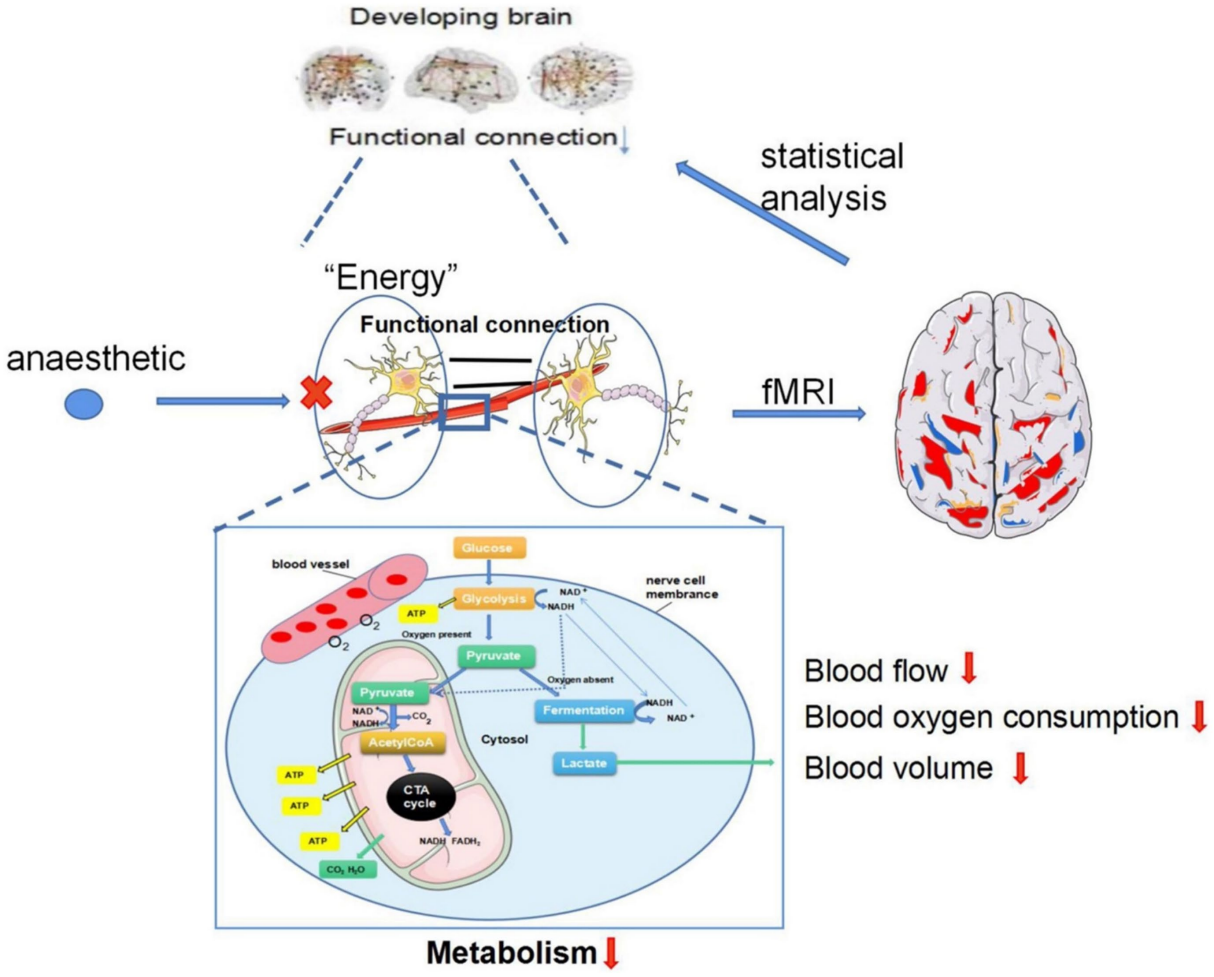
The speculated mechanism of fMRI recording the anesthetics alter functional connections in the developing brain. (1) Neurons operate autonomically in resting state, the brain provides energy for neurons through glycolysis. Images of resting state brain can be obtained through fMRI and functional connections of resting state brain can be obtained through statistical analysis. (2) Anesthesia exposure can lead to decreased the blood flow, oxygen consumption, and blood volume of neurons, which affects the glycolysis process of the brain, resulting in decreased energy of neurons, reduced BOLD signal captured by fMRI, and decreased functional connectivity (17).

Both Global Neuronal Workspace Theory (GNWT) and the Integrated Information Theory (IIT) converge on the notion that anesthetic-induced unconsciousness arises from disrupted large-scale information integration rather than complete neuronal silence. These models collectively offer conceptual frameworks to elucidate the mechanisms by which anesthetic agents disrupt neural communication, resulting in drug-induced unconsciousness while preserving variable residual activity in sensory or motor circuits (14–16). Figure 2 shows the patterns of connectivity in the distributed neural network that are involved in consciousness. It also shows how anesthetic agents interfere with thalamocortical and frontoparietal integration when a person is unconscious (17). It is important to note that Figure 2 specifically depicts mechanisms in the developing brain, which exhibits distinct metabolic and connectivity characteristics compared to mature adult brains. While these developmental studies provide foundational insights into anesthetic effects on neural circuits, direct extrapolation to adult perioperative consciousness requires caution, as mature brains possess established network architectures and different neuroplasticity profiles.

At the molecular level, the modulation of consciousness induced by anesthetics is regulated by interactions at the receptor level. Drugs like propofol and sevoflurane mainly make inhibitory GABA_A receptors stronger, which makes cortical neurons hyperpolarized and makes them fire less. Ketamine and nitrous oxide, on the other hand, block NMDA receptors, which stops excitatory glutamatergic transmission and makes it harder for the cortex to integrate information. Dexmedetomidine works by acting on α2-adrenergic receptors, which is like how natural sleep pathways work. It does this by blocking the locus coeruleus and lowering noradrenergic tone (4, 16, 18). Despite the differences in these pharmacologic actions, they ultimately lead to the same result: a decrease in large-scale neural coherence and a breakdown in the flow of information within cortical hierarchies. This diversity of molecular pathways demonstrates that drug-induced unconsciousness is not a unitary brain state, but rather a spectrum of neurophysiological configurations characterized by varying degrees of neural disconnection, inhibitory dominance, and network disorganization.

Neuroimaging and electrophysiological studies have provided additional insights into the brain’s dynamic transitions during anesthesia. Functional MRI analyses demonstrate that during loss of behavioral responsiveness, connectivity within the default mode network (DMN) and frontoparietal control network significantly diminishes, whereas local synchronization within sensory regions frequently endures. Electroencephalographic (EEG) studies reveal a transition to lower-frequency oscillations, characterized by alpha and delta dominance, which signifies profound anesthetic states. At deeper levels, burst suppression patterns emerge—alternating high-voltage bursts with periods of electrical silence—representing profound cortical inactivation. This pattern serves as a critical biomarker for excessive anesthetic depth and is strongly associated with postoperative delirium and cognitive dysfunction, particularly in elderly patients (19, 20). Notably, this relationship holds across all anesthesia settings except cardiac anesthesia, where hypothermia and anesthetic management intentionally induce burst suppression as a desired neuroprotective strategy (21). Nonetheless, notwithstanding their clinical applicability, these metrics yield only indirect estimates, as consciousness cannot be exclusively attributed to electrical activity but encompasses intricate, emergent characteristics of neural organization.

Network-level analyses further support the view that anesthetic unconsciousness reflects reconfigured connectivity states, a concept that underpins emerging predictive monitoring approaches discussed later (22–25).

These insights have direct implications for perioperative management in a clinical setting. A more thorough understanding of how anesthetics change the way the brain works as a whole can help create personalized dosing plans that lower the chance of under-sedation (which can cause awareness during surgery) or over-suppression (which can cause delayed emergence and postoperative delirium). It also makes it possible to use targeted neuroprotective treatments, like changing inflammation or oxidative stress that could slow down the recovery of the nervous system after anesthesia.

## 4 Anesthetic pharmacodynamics and consciousness modulation

The pharmacodynamic mechanisms by which anesthetic agents modify states of consciousness involve a sophisticated interaction among molecular targets, neuronal circuits, and extensive brain networks. The suppression of consciousness during anesthesia is not attributable to a singular biochemical occurrence; instead, it arises from synchronized inhibition and disruption across various neurotransmitter systems. Intravenous, volatile, and dissociative anesthetics all work by blocking different receptor pathways that send signals between neurons. However, they all have the same effect: they make the brain less connected and less responsive. Comprehending these pharmacodynamic interactions is essential for elucidating the origins of perioperative consciousness disturbances and their potential prevention via tailored anesthetic approaches (26–28).

At the molecular level, general anesthetics primarily function by modulating synaptic transmission via ligand-gated ion channels. Intravenous agents like propofol, thiopental, and etomidate work by increasing the transmission of inhibitory GABAergic signals. This mechanism lessens the excitability of the cortex and messes up thalamocortical signaling, which is very important for being aware of things. Likewise, volatile anesthetics like sevoflurane and isoflurane enhance GABAergic inhibition and inhibit excitatory glutamatergic transmission via NMDA receptor antagonism. The resulting net inhibitory effect diminishes neuronal synchronization in cortical and subcortical areas, leading to a state of regulated drug-induced unconsciousness marked by low-frequency oscillatory dominance in EEG recordings.

Ketamine, on the other hand, is an unusual drug among anesthetics. As a strong NMDA receptor antagonist, it puts the body in a dissociative anesthetic state where sensory processing keeps going but perceptual integration stops. This contradictory arrangement shows that consciousness is not a simple on–off switch, but a complex system that can be broken in many ways. During ketamine anesthesia, EEG and imaging studies indicate heightened frontal high-frequency activity, implying maintained or potentially augmented local processing despite global disconnection. These findings highlight that anesthetic modulation of consciousness can occur through various neurophysiological pathways (some prioritizing inhibition, while others focus on disintegration) (1, 29, 30).

The adrenergic system is also very important for how anesthetics work. Dexmedetomidine, a selective α2-adrenergic receptor agonist, induces a sedative state that closely resembles natural sleep by inhibiting activity in the locus coeruleus and decreasing norepinephrine release. Dexmedetomidine preserves specific thalamocortical interactions, resulting in a lighter and more physiologically coherent state of drug-induced unconsciousness, in contrast to GABAergic anesthetics. This characteristic elucidates its increasing clinical application for conscious sedation and neuroprotective anesthesia. Opioid-based adjuncts like fentanyl and remifentanil also help change consciousness by activating *μ*-opioid receptors, which lowers ascending nociceptive input and indirectly lowers cortical arousal and sensory awareness. Combining opioids with hypnotic agents improves hemodynamic stability and lets you lower the dose, which lowers the risk of cognitive impairment after surgery (31–33).

*At the systems level, anesthetic pharmacodynamics alter the connectivity architecture established in Section 2, diminishing long-range coherence within networks critical for conscious integration.* The extent of connectivity loss is associated with anesthetic depth, serving as a quantifiable biomarker for drug-induced unconsciousness. However, too much suppression of cortical dynamics can make recovery harder and lead to postoperative delirium, especially in older people or those with neurological problems. Consequently, it is essential to preserve an optimal pharmacodynamic equilibrium—adequate to induce drug-induced unconsciousness without disrupting cognitive networks (34–36).

The pharmacokinetic–pharmacodynamic (PK–PD) relationship makes it even harder to guess how an anesthetic will work. Differences between people in how their livers break down drugs, how well plasma proteins bind to drugs, and how easily drugs cross the blood–brain barrier all affect how well anesthetics work on neural targets. Genetic polymorphisms in enzymes like CYP2B6 and GABRA1 can change how sensitive a person is to drugs. This is why some people become aware of their surroundings unexpectedly or take longer to wake up. Additionally, physiological states such as hypoxia, acidosis, or systemic inflammation can alter receptor functionality and neurotransmitter release, thereby affecting the anesthetic–consciousness interface. This variability has led to more interest in personalized anesthesia, which tries to use genomic, metabolic, and electrophysiological data to customize anesthetic dosing for each patient (37, 38).

New pharmacodynamic models are using more and more computer and artificial intelligence systems to guess how anesthetics will work. AI-driven systems can figure out how likely it is that someone will become aware of something by looking at EEG signatures, hemodynamic variables, and drug infusion parameters. They can also suggest changes in real time to keep the depth of anesthesia at the right level. Machine learning models, especially deep neural networks, have shown good accuracy in linking EEG patterns caused by drugs to states of consciousness. These changes mean that we are moving toward closed-loop anesthesia systems, which automatically adjust the delivery of drugs based on neurophysiological feedback. This reduces the risks of both intraoperative awareness and too much sedation.

The pharmacodynamic effects of anesthetic agents persist beyond the surgical environment, influencing neural recovery in the postoperative phase. Extended exposure to specific agents, particularly in elderly patients, may result in neuroinflammation, synaptic remodeling, and mitochondrial dysfunction, thereby exacerbating postoperative cognitive decline. On the other hand, drugs like dexmedetomidine and xenon protect the brain by stopping inflammatory cascades and making synapses more stable. Ongoing research continues to investigate the molecular pathways through which anesthetics influence neural plasticity, with the objective of identifying pharmacological strategies that maintain both the integrity of consciousness and cognitive resilience (39–42).

Target-Controlled Infusion (TCI) systems use computer algorithms to achieve precise drug concentrations for Total Intravenous Anesthesia (TIVA) based on patient-specific parameters, enabling accurate titration that reduces awareness and overdose risks (43). Advantages include stable delivery, faster recovery, and EEG integration. However, limitations persist in special populations (obesity, organ dysfunction, pediatrics) due to pharmacokinetic variability. The propofol-remifentanil combination is optimal, with typical effect-site concentrations of 2–6 μg/mL (propofol) and 2–8 ng/mL (remifentanil). Processed EEG monitoring (BIS/entropy 40–60) reduces awareness risk by 60–80% in high-risk surgeries (44). Emerging personalized algorithms incorporating real-time EEG feedback promise enhanced safety and efficacy.

In essence, anesthetic pharmacodynamics signify a nuanced balance between neural suppression and the maintenance of systemic equilibrium. Every drug alters consciousness via distinct receptor mechanisms, yet collectively leads to a singular neurophysiological effect (transient interruption of global information exchange). Nonetheless, the identical mechanisms that inhibit consciousness can, in dysregulated circumstances, result in perioperative disturbances. A more profound understanding of these molecular and systemic interactions constitutes the scientific foundation for the subsequent examination of intraoperative awareness and postoperative cognitive dysfunction, the two most clinically significant indicators of consciousness disruption in anesthetic practice.

While both total intravenous anesthesia (TIVA) and inhalational anesthesia achieve reversible drug-induced unconsciousness via distinct delivery modalities, their differential impacts on perioperative disturbances of consciousness and cognition warrant detailed comparison (Table 1). In contemporary practice, pure inhalational anesthesia is rarely used; rather, inhalational agents are administered as part of a balanced technique incorporating opioids and adjuncts. This distinction is crucial for clinical decision-making, particularly regarding intraoperative awareness and postoperative cognitive trajectories.

**Table 1:** Comparative analysis of TIVA and inhalational anesthesia across key domains.

Feature	Total intravenous anesthesia (TIVA)	Inhalational anesthesia (e.g., sevoflurane, isoflurane)
Primary mechanism	Potentiation of GABA_A receptors (propofol) + opioid-mediated analgesia	GABA_A potentiation + NMDA antagonism + modulation of two-pore domain potassium channels
Pharmacokinetic control	Precise titration via target-controlled infusion (TCI); rapid context-sensitive half-time adjustment	Dose-dependent alveolar concentration (MAC) control; slower emergence due to tissue accumulation
Intraoperative awareness risk	Higher risk during equipment failure (pump malfunction, IV disconnection) or PK variability; requires vigilant monitoring of plasma effect-site concentration	Lower equipment-dependent risk; end-tidal concentration monitoring provides direct dose verification
EEG signature	Predominant alpha oscillations (8–12 Hz) with dose-dependent burst suppression; BIS typically 40–60	Theta/delta dominance; dose-dependent frontal alpha coherence; BIS reliability may vary with agent
Emergence profile	Faster, more predictable emergence due to rapid redistribution; reduced postoperative delirium incidence in meta-analyses	Slower emergence; risk of “phase II” block with accumulation in fat/muscle; higher PONV incidence
Neuroinflammatory impact	Propofol exhibits antioxidant properties; lower IL-6/TNF-α elevation; neuroprotective in ischemic models	Volatile agents may exacerbate neuroinflammation and amyloid-β oligomerization; potential cognitive toxicity
Clinical vulnerability	Increased risk in obese patients (altered volume of distribution) and chronic opioid users (tolerance)	Risk of environmental pollution; contraindicated in malignant hyperthermia-susceptible patients
Cost & accessibility	Higher drug cost; requires specialized infusion pumps; steeper learning curve	Lower cost; widely available; familiar administration; requires scavenging systems
Hemodynamic effects	Propofol causes dose-dependent vasodilation and myocardial depression; risk of hypotension; generally preserves autonomic tone	Dose-dependent hypotension via peripheral vasodilation and reduced cardiac contractility; potential tachycardia with isoflurane; generally well-tolerated in balanced technique with opioid co-administration
Cardioprotective properties	Minimal direct cardioprotective effects; some antioxidant properties	Well-documented cardioprotection via ischemic preconditioning mechanisms; reduced infarct size and improved outcomes in cardiac surgery

MAC, minimum alveolar concentration; PONV, postoperative nausea and vomiting; PK, pharmacokinetic.

## 5 Intraoperative awareness and explicit recall

Intraoperative awareness—defined as conscious recollection of events during intended anesthetic-induced unresponsiveness—remains a distressing complication. Even though anesthetic techniques and monitoring systems have come a long way, the phenomenon still happens at a low but clinically important rate. When this fragile balance fails, the resulting awareness can lead to lasting neuropsychological effects, from temporary anxiety to chronic post-traumatic stress disorder (PTSD). Comprehending the neurophysiological, pharmacological, and systemic factors that influence intraoperative awareness is essential for the formulation of preventive strategies and the enhancement of perioperative care (45–48).

Awareness arises when anesthetic-induced suppression of cortical integration is insufficient to counteract surgical stimulation, particularly under conditions requiring anesthetic dose limitation (49–51). The neurobiology of intraoperative awareness entails the partial inhibition of thalamocortical communication and the persistence of sensory pathway activation. Research employing electroencephalography (EEG) and positron emission tomography (PET) reveals that during awareness events, activity in the auditory cortex and associated areas persists, signifying intact sensory processing. Nonetheless, the distinguishing factor between these events and unconscious perception is the reestablishment of connectivity between sensory cortices and the prefrontal executive network, facilitating memory encoding and conscious recognition. Incomplete suppression of the hippocampal and amygdalar circuits enhances emotional memory formation, elucidating the frequent association of awareness experiences with intense fear or panic (52).

In a clinical setting, intraoperative awareness can be divided into explicit and implicit types. Explicit awareness denotes conscious recollection of intraoperative occurrences postoperatively, whereas implicit awareness entails subconscious perception devoid of recollection, yet potentially influencing postoperative behavior or emotional responses. Both types share neural pathways that connect the limbic system and networks that make the cortex more active (53–55).

Intraoperative awareness is associated with a convergence of anesthetic, patient-related, and surgical factors that compromise adequate suppression of cortical integration. Clinically, risk is increased when anesthetic delivery is intentionally or unintentionally limited, such as during neuromuscular blockade, total intravenous anesthesia with pharmacokinetic variability, or procedures requiring hemodynamic restraint, as well as in patients with heightened anesthetic requirements or vulnerability (56, 57). Beyond its immediate intraoperative occurrence, awareness may lead to persistent psychological sequelae, ranging from acute distress to long-term post-traumatic stress symptoms, particularly when experiences involve pain, paralysis, or fear. These consequences underscore the importance of both preventive intraoperative strategies and structured postoperative assessment and support for affected patients.

EEG-based monitoring may detect surrogate markers of insufficient anesthetic suppression, although predictive accuracy remains limited (58, 59). Preventive strategies stress personalized anesthetic titration, careful equipment checks, and clear communication among surgical teams in addition to technological fixes. Taking amnestic drugs like benzodiazepines before induction may make it harder to remember things, but they do not stop people from being aware during surgery. Structured awareness reporting systems, such as the Brice questionnaire, aid in the postoperative identification and enable a systematic evaluation of awareness incidence. Importantly, open disclosure and psychological support following suspected awareness events have been demonstrated to alleviate long-term emotional distress (60, 61).

## 6 Postoperative delirium and cognitive dysfunction

### 6.1 Overview and clinical significance

Perioperative Neurocognitive Disorders (PND) represent the most common neuropsychiatric complications that occur after anesthesia and surgery, especially in older and very sick patients. Within this framework, postoperative delirium (POD) and cognitive dysfunction following surgery are key manifestations that indicate disturbances in neural connectivity and neurotransmission during the perioperative period, frequently presenting as variable attention, disorganized cognition, or compromised memory. Delirium generally manifests acutely within the initial 48–72 h post-surgery, whereas cognitive impairment (classified as Delayed Neurocognitive Recovery when lasting up to 30 days, or Postoperative Neurocognitive Disorder when persisting beyond 30 days) may endure for weeks or months, impeding rehabilitation and long-term cognitive efficacy. The increasing global surgical demographic highlights the necessity of comprehending their common and unique neurobiological underpinnings within anesthesiology (62–64).

### 6.2 Neuroinflammatory and cellular mechanisms

Neuroinflammation is a major contributor to postoperative cognitive dysfunction. Surgical trauma and anesthesia initiate systemic inflammatory cascades, increasing cytokines like IL-1β, TNF-*α*, and IL-6, which breach the blood–brain barrier (BBB) and interfere with neuronal communication. This process stimulates microglia and astrocytes, resulting in oxidative stress and synaptic impairment in critical cognitive areas, including the hippocampus and prefrontal cortex. Animal studies demonstrate that heightened microglial activity is associated with sustained neurocognitive deficits, whereas pharmacological inhibition of inflammatory mediators alleviates these impairments. These findings indicate that anesthesia-induced immune activation serves a dual function (promoting recovery while rendering individuals susceptible to transient or chronic delirium) (65–68).

### 6.3 Neurotransmitter imbalance and network disruption

The neurotransmitter landscape of the postoperative brain undergoes profound alterations, with cholinergic deficiency and dopaminergic hyperactivity being primary contributors to delirium pathogenesis. Lower levels of acetylcholine make it harder for the brain to wake up and pay attention, while higher levels of dopamine mess up sensory gating and thought organization. Anesthetic agents also change the GABAergic and glutamatergic pathways, which makes the balance between excitatory and inhibitory even worse. Functional MRI studies show that the default mode and frontoparietal networks break down for a short time, which is similar to what happens in the mind when it is fragmented. These disturbances underscore that delirium and POCD are not discrete disorders but rather dynamic manifestations of compromised neural communication across distributed brain systems (69).

### 6.4 Role of anesthetic agents and surgical factors

Different anesthetic agents have different effects on neurocognitive outcomes after surgery. Volatile anesthetics, including isoflurane and sevoflurane, have been correlated with increased neuroinflammatory markers and amyloid precursor protein expression, indicating possible associations with neurodegenerative risk and increased incidence of Postoperative Neurocognitive Disorder. On the other hand, intravenous drugs like propofol and dexmedetomidine have better neuroprotective profiles because they reduce inflammation and keep mitochondria healthy. Surgical factors, including duration, blood loss, and hypoxia, also play a major role. Long-term anesthesia raises oxidative stress, and low blood pressure and microemboli make it harder for blood to flow to the brain. Multimodal anesthesia techniques that reduce exposure to neurotoxic doses and combine analgesia with sedation are being recommended more and more to lower these risks (70).

### 6.5 Cognitive and behavioral manifestations

Clinically, postoperative delirium manifests as fluctuating attention, visual hallucinations, disorientation, and emotional lability, frequently intensifying at night due to circadian rhythm disruption. Postoperative cognitive dysfunction (now termed Delayed Neurocognitive Recovery or Postoperative Neurocognitive Disorder depending on duration) (71), on the other hand, shows up in small ways, like forgetting things, taking longer to process information, or having trouble with executive functions. These symptoms may not be noticed until normal activities resume. Objective neuropsychological testing encompassing memory, attention, and psychomotor domains is crucial for detection, especially in older adults or individuals with preexisting cognitive impairment. Persistent POCD is linked to a lower quality of life, longer hospital stays, and a higher risk of death in the long term, which shows how important it is to know what will happen (72).

### 6.6 Emerging predictive markers and diagnostic tools

Finding predictive biomarkers for postoperative cognitive disturbances is a growing area of focus in contemporary research. Neural damage and cognitive decline are associated with elevated serum levels of neuron-specific enolase (NSE), S100β, and inflammatory cytokines. Even in patients with clinically mild symptoms, white matter microstructural changes can be detected by neuroimaging modalities like diffusion tensor imaging (DTI). Furthermore, machine learning models that incorporate perioperative physiological data and EEG-based delirium indices have demonstrated promise in forecasting cognitive trajectories. Targeted neuroprotective interventions and customized anesthetic planning are made possible by early identification of at-risk individuals (73).

### 6.7 Prevention and neuroprotective strategies

Preventive strategies focus on reducing neuroinflammation, keeping cerebral perfusion steady, and keeping the balance of neurotransmitters stable. Monitoring oxygenation and hemodynamics during surgery lowers the risk of ischemia, and avoiding anticholinergic drugs keeps neurotransmitters from running out. Dexmedetomidine infusion has shown effectiveness in reducing the incidence of delirium due to its anti-inflammatory and sleep enhancing characteristics. Cognitive prehabilitation programs, which include memory training, sleep optimization, and nutritional support, make people even more resilient to cognitive decline after surgery. The combination of pharmacologic and behavioral strategies signifies a transformative shift towards comprehensive perioperative neuroprotection (74).

## 7 Diagnostic and monitoring technologies

### 7.1 Evolution of anesthetic monitoring

Over the past twenty years, advances in neuroimaging, electrophysiology, and computational analysis have made it much easier to keep track of the depth of anesthesia and notice when someone wakes up. Modern anesthesiology is using brain-based monitoring systems more and more to keep an eye on neural activity in real time. The goal is to measure consciousness using physiological signals instead of subjective clinical interpretation (75).

### 7.2 Electroencephalography and derived indices

Electroencephalography (EEG) remains the primary modality for real-time assessment of anesthetic depth, as it directly reflects cortical activity and network synchronization. Anesthetic agents induce characteristic spectral changes, including shifts toward lower-frequency oscillations and, at excessive depths, the emergence of burst suppression patterns. Processed EEG indices such as the bispectral index, entropy, and patient state index translate complex signals into simplified numerical values, facilitating intraoperative interpretation. However, these indices provide indirect surrogates of consciousness and are influenced by drug-specific EEG signatures, neuromuscular blockade, hypothermia, and signal artifacts. Increasing attention has therefore shifted from scalar thresholds to pattern-based EEG interpretation, recognizing that transitions in consciousness are better captured by dynamic spectral and spatial features. While EEG-based monitoring has improved patient safety, its capacity to reliably predict awareness or cognitive outcomes remains limited, underscoring the need for cautious clinical interpretation (76).

DSA pattern recognition may outperforms BIS for detecting consciousness transitions. Loss of behavioral responsiveness shows fronto-central alpha oscillations; intraoperative awareness manifests as desynchronization or lost alpha spindles (77). Machine learning now classifies these patterns predictively, enabling pre-behavioral awareness detection (78). This shift from threshold alerts to integrated spectral pattern analysis represents the next frontier in precision anesthesia.

### 7.3 Functional neuroimaging and network connectivity

**Figure 3:**
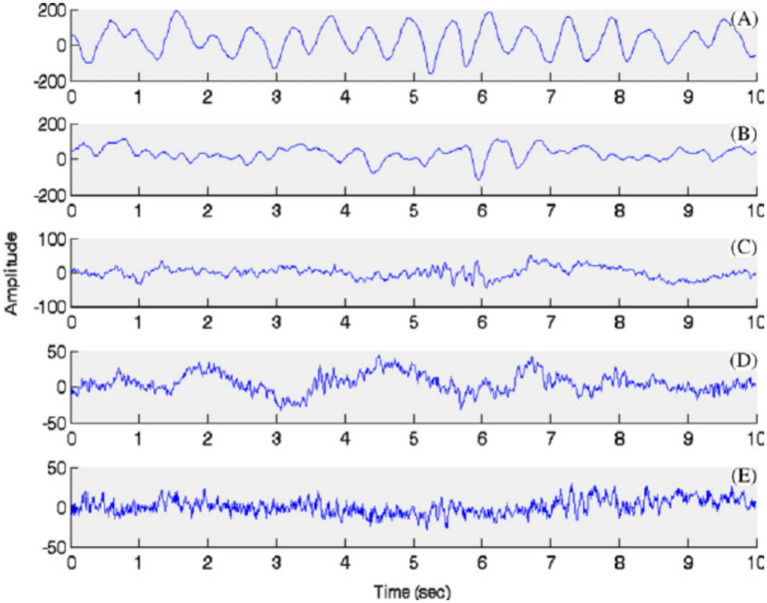
Selected EEG time series from different anesthetic depths under propofol anesthesia. In **(A)** (BIS 15), the trace depicts a burst suppression pattern, characterized by alternating periods of high-amplitude bursts and isoelectric silence, indicative of very deep anesthesia. **(B–E)** Show BIS indices of 30, 50, 80, and 97, corresponding to deep, moderate, light anesthesia and awake states, respectively (79). Note that EEG signatures vary significantly across different anesthetic agents.

Functional neuroimaging techniques, including functional magnetic resonance imaging (fMRI) and positron emission tomography (PET), have substantially advanced mechanistic understanding of anesthetic-induced unconsciousness. These modalities consistently demonstrate reduced connectivity within large-scale networks, particularly involving thalamocortical and frontoparietal systems, alongside decreased metabolic activity in higher-order association cortices. Much of this evidence derives from preclinical or perioperative resting-state studies, as real-time intraoperative imaging in humans remains constrained by ethical and logistical limitations. Consequently, functional imaging serves primarily as a translational research tool rather than a clinical monitoring modality. Nonetheless, insights gained from network-level imaging have informed interpretation of electrophysiological signatures and contributed to the identification of biomarkers associated with anesthetic depth and emergence trajectories. These findings provide an essential mechanistic foundation for the development of future portable and network-informed monitoring technologies (79) (Figure 3).

### 7.4 Artificial intelligence and predictive analytics

Artificial intelligence (AI) has emerged as an exploratory approach for integrating multimodal physiological data to support anesthetic monitoring. Machine learning models combining EEG features, hemodynamic variables, and pharmacokinetic information have shown promise in classifying states of responsiveness and identifying patterns associated with excessive anesthetic depth, particularly burst suppression. However, most reported performance metrics originate from retrospective or single-center datasets, and prospective validation of real-time awareness prediction remains limited. Closed-loop anesthesia systems have been proposed as an extension of this concept, using algorithmic feedback to adjust drug delivery based on neurophysiological signals. While such systems may enhance dosing consistency, their clinical impact, generalizability, and ethical implications require further evaluation. Within the proposed framework, AI-based analytics should be regarded as adjunctive tools that augment, rather than replace, clinician judgment in managing perioperative disturbances of consciousness and cognition (80).

### 7.5 Multimodal integration and future directions

The future of perioperative consciousness monitoring depends on combining different types of data into single analytic frameworks. Integrating EEG, fNIRS, and hemodynamic data facilitates a thorough evaluation of both cortical and subcortical processes. Hybrid monitoring systems can detect patterns of residual awareness or imminent emergence prior to the manifestation of clinical signs. Moreover, progress in wearable neurotechnology and wireless sensors is facilitating the extension of monitoring into the postoperative phase, allowing for ongoing assessment of cognitive recovery. This convergence of technology, neuroscience, and computational modeling is gradually redefining anesthesiology as a data-driven discipline, wherein consciousness is not only observed but quantitatively analyzed and predictively regulated. Table 2 provides a conceptual summary of the multifactorial landscape of perioperative consciousness disturbances, incorporating neural, pharmacologic, inflammatory, and diagnostic domains, along with their principal mechanistic dimensions and associated clinical implications.

**Table 2:** Summary.

Domain	Core mechanisms/Features	Representative anesthetic or pathophysiological factors	Clinical implications
Neurobiological (23)	Disruption of thalamocortical and frontoparietal network connectivity; reduced global integration	Suppression of cortical gamma oscillations by propofol, sevoflurane	Induction and maintenance of unconsciousness; potential incomplete suppression leading to awareness
Pharmacodynamic (38)	Modulation of GABAergic and NMDA receptor pathways; inhibition of excitatory neurotransmission	GABA_A receptor potentiation, NMDA receptor antagonism	Depth-dependent consciousness alteration; dose variability linked to emergence phenomena
Neuroinflammatory (67)	Cytokine release and microglial activation impairing neural signaling and cognition	Elevated IL-6, TNF-α following surgery and anesthesia	Development of postoperative delirium and cognitive dysfunction
Monitoring & diagnostics (59)	Quantitative EEG, BIS, fNIRS, AEPs, AI-driven models for anesthetic depth	BIS < 60 indicates adequate sedation; multimodal integration improves accuracy	Real-time detection of awareness and optimization of anesthetic titration
Therapeutic/preventive (74)	Anti-inflammatory modulation, precision dosing, multimodal sedation strategies	Dexmedetomidine, xenon, closed-loop delivery systems	Reduction in delirium incidence and improved postoperative cognitive recovery

Modern TCI systems integrate AI-driven predictive models analyzing drug effect-site concentrations, real-time EEG parameters, hemodynamic variables, and patient-specific genetic profiles. This multimodal fusion enables dynamic adjustment of target concentrations based on predicted individual responses rather than population averages. Next-generation closed-loop TIVA systems automatically titrate propofol and remifentanil infusions to maintain optimal effect-site concentrations while preserving BIS/entropy targets within narrow therapeutic windows (6). These intelligent platforms incorporate safety algorithms detecting and compensating for sudden surgical stimulation, hemodynamic instability, or equipment malfunction, representing a significant leap toward fully automated, personalized anesthesia delivery.

## 8 Therapeutic and preventive strategies

### 8.1 Pharmacological modulation and dose optimization

The foundation of averting perioperative disturbances of consciousness and cognition is meticulous pharmacological regulation. Customizing anesthetic delivery to each person’s neurophysiological responses reduces both intraoperative awareness and cognitive problems after surgery. Agents like propofol and sevoflurane are adjusted based on depth-monitoring indices. Opioids and benzodiazepines, on the other hand, help keep amnesia and pain relief without too much cortical suppression. Recent studies underscore the advantages of balanced anesthesia, which integrates various drug classes to induce hypnosis, analgesia, and muscle relaxation via synergistic mechanisms. Dexmedetomidine has also become well-known for its sedative, anxiolytic, and anti-inflammatory effects. It causes a sleep-like sedation pattern while lowering the risk of delirium. Recommended dosing protocols (loading dose 0.5–1.0 μg/kg over 10 min, followed by infusion 0.2–0.7 μg/kg/h) optimize efficacy while minimizing bradycardia and hypotension. Its use is particularly beneficial in elderly patients (>65 years) and those with preexisting cognitive impairment, where it can replace GABAergic agents to reduce POD risk by maintaining physiological sleep–wake cycles (81). Enhancing pharmacological synergy via multimodal regimens signifies a pivotal advancement in neuroprotective anesthesia (74).

### 8.2 Anti-inflammatory and neuroprotective interventions

Neuroinflammation continues to be a significant factor in postoperative delirium and cognitive impairment; thus, targeting inflammatory pathways is an essential therapeutic goal. Perioperative administration of agents such as nonsteroidal anti-inflammatory drugs (NSAIDs), corticosteroids, and statins has been shown to diminish systemic cytokine responses that impair neural function. Dexmedetomidine’s α2-adrenergic action also helps reduce neuroinflammation by stopping microglial activation and lowering oxidative stress. Specifically, it suppresses NF-κB signaling pathway activation, thereby downregulating COX-2 and iNOS expression. This reduces production of reactive oxygen species (ROS) and lipid peroxidation in hippocampal neurons, preserving synaptic plasticity-related proteins (e.g., BDNF, PSD-95) critical for cognitive recovery post-surgery. Experimental research investigates xenon and lidocaine for their neuroprotective properties, as both substances regulate NMDA receptor activity and maintain mitochondrial stability. These interventions are designed to reduce neuronal damage during anesthesia and facilitate expedited cognitive recovery and stable postoperative neural reintegration (74).

### 8.3 Non-pharmacological and cognitive strategies

Non-pharmacological interventions have become crucial adjuncts to pharmacological strategies in mitigating perioperative consciousness disturbances. Preoperative cognitive screening helps find people who are likely to have delirium after surgery, so that appropriate care can be given. Controlling light exposure and reducing sleep disruption to keep normal circadian rhythms significantly improves postoperative orientation. Early mobilization, hydration, and sensory reorientation diminish the incidence of delirium, especially among older patients. Cognitive training programs, such as memory and attention exercises conducted before surgery, enhance neural plasticity and resilience. Structured communication among surgical teams also makes sure that everyone knows the depth of anesthesia and the hemodynamic parameters in real time, which reduces the chance of human error in anesthetic titration (23).

### 8.4 Intraoperative neuromonitoring and closed-loop systems

Continuous neuromonitoring is still very important for both prevention and treatment. Real-time EEG-based indices, auditory evoked potentials, and functional near-infrared spectroscopy (fNIRS) offer dynamic insights into cortical suppression and recovery, facilitating immediate adjustments to anesthetic dosing. The combination of these technologies into closed-loop anesthesia delivery systems is a big step toward precise control. In these systems, machine learning algorithms automatically adjust the flow of anesthetic based on neurophysiological feedback, keeping the right level of sedation without the need for a doctor to be present. This feedback-driven method has shown potential for keeping people unconscious and may stopping them from becoming too sedated, which is a big cause of cognitive problems after surgery. These smart systems are always getting better, which means that future anesthetic care will be safer and more reliable (41).

### 8.5 Personalized anesthetic planning

Personalized or precision anesthesia combines genomic, metabolic, and neurophysiological information to direct tailored anesthetic management. Differences in genes that code for GABA and NMDA receptor subunits, as well as differences in drug-metabolizing enzymes like CYP2B6, can affect how sensitive and long-lasting anesthetics are. Using this kind of information to make decisions about surgery lets doctors choose the best drugs and doses for each patient. Personalized anesthesia also takes into account patients’ other health problems, mental health, and previous exposure to anesthetics. Artificial intelligence-assisted clinical platforms are being created to combine these multidimensional datasets. These platforms will be able to predict the risks of anesthesia and cognitive outcomes. This change from standard protocols to precision-guided anesthesia is a big step toward neuroprotection that puts the patient first (48).

## 9 Conclusion

Understanding perioperative consciousness disorders represents a turning point in modern anesthesiology. It brings together neurobiology, pharmacology, and computational science into one field of study. Consciousness, previously regarded as an abstract and unquantifiable phenomenon, is now increasingly recognized as an emergent property of dynamic brain networks—networks that anesthetic agents modulate with remarkable precision yet occasional unpredictability. Anesthesiology has evolved from an art of sedation to a science of controlled neural modulation by examining intraoperative awareness, postoperative delirium, and cognitive dysfunction. The goal is not only to induce drug-induced unconsciousness but also to maintain the integrity of the subsequent recovery.

Recent progress in molecular and cellular neuroscience has demonstrated that anesthesia does not merely inhibit brain activity; it reorganizes its connectivity. The targeted inhibition of thalamocortical pathways, the suppression of excitatory neurotransmission, and the enhancement of inhibitory networks collectively alter neural synchrony to induce reversible drug-induced unconsciousness. Nonetheless, this identical plasticity that facilitates reversible suppression simultaneously engenders vulnerability, permitting consciousness to re-emerge prematurely or to recover in an aberrant manner. The ongoing prevalence of perioperative consciousness disturbances, notwithstanding advancements in technology, highlights that consciousness cannot be solely regulated by pharmacology. Instead, it is a fluid continuum that is affected by molecular biology, systemic physiology, and the resilience of each person’s neurons.

The combination of neuroimaging and electrophysiological tools has changed how we understand and keep track of the depth of anesthesia. EEG-derived indices, functional MRI, and near-infrared spectroscopy have illuminated brain activity during anesthesia, allowing clinicians to correlate physiological parameters with subjective awareness. Artificial intelligence has enhanced these abilities by converting intricate neural data patterns into predictive models that can detect early deviations indicative of consciousness. These new ideas point to a future where anesthetic administration will be dynamically controlled by feedback systems that constantly read brain signals, making sure that there is a balance between drug-induced unconsciousness and neuroprotection. However, this automation must coexist with clinical intuition, as the human ability for contextual judgment is indispensable in navigating the complexities of consciousness.

Pharmacological innovation is progressing towards targeted modulation instead of broad suppression. The advancement of anesthetic agents that replicate natural sleep architecture, exemplified by dexmedetomidine, signifies a comprehensive paradigm shift towards physiological coherence rather than profound suppression. The increasing recognition of neuroinflammation and oxidative stress as factors in postoperative cognitive dysfunction has expanded therapeutic approaches beyond sedation, promoting the creation of adjunct agents with anti-inflammatory and mitochondrial-stabilizing effects. These discoveries have laid the foundation for a new generation of anesthetic protocols focused on neural preservation rather than mere immobility.

Anesthesiology now focuses on personalized care at the clinical level. This means taking into account genetic, metabolic, and psychological differences when planning anesthesia. Personalized anesthesia, bolstered by pharmacogenomic data and ongoing neurophysiological monitoring, mitigates the risks associated with both underdosage, which can lead to awareness, and overdosage, which may cause delayed emergence or delirium. This precision-based approach signifies a significant philosophical transition from standardized protocols to adaptive medicine, wherein each patient’s neural profile informs therapeutic choices. This kind of personalization brings anesthesiology in line with the bigger trend in medicine toward care that is based on data and focused on the patient.

Even with these scientific advances, perioperative consciousness is still a field where technology and human experience meet. Awareness during anesthesia, although infrequent, possesses psychological significance that surpasses physiological constraints. The distress experienced by patients who regain consciousness during surgery highlights the profoundly ethical aspect of anesthesia practice. It calls for care that includes compassion, open communication, and psychological follow-up. The future of anesthesiology hinges not solely on computational accuracy but also on the reaffirmation of humanistic principles that regard consciousness as the fundamental essence of personhood.

The future of anesthetic science will probably be characterized by integration, combining artificial intelligence, neuroimaging, pharmacogenomics, and behavioral neuroscience into cohesive predictive frameworks. The ultimate goal of perioperative neuroprotection is to be able to predict changes in consciousness, anticipate cognitive outcomes, and customize treatments for each patient’s neural architecture. Ongoing research into network-level modeling, biomarker identification, and closed-loop anesthetic systems will progressively reduce the margin for error in consciousness management. As anesthesiology approaches this frontier, it exists not only as a procedural specialty but as a discipline central to human neuroscience—where the mastery of consciousness delineates the scientific and ethical parameters of contemporary medicine.
